# Evaluating the utility of an immune checkpoint-related lncRNA signature for identifying the prognosis and immunotherapy response of lung adenocarcinoma

**DOI:** 10.1038/s41598-022-16715-0

**Published:** 2022-07-27

**Authors:** Hongpan Zhang, Meihan Liu, Zhihao Yang, Guobo Du, Bin Yu, Yan Gui, Lu Cao, Xianfu Li, Bangxian Tan

**Affiliations:** 1 Department of Oncology, Affiliated Hospital of North Sichuan Medical College, No. 1, Maoyuan south road, Shunqing District, Nanchong City, Sichuan Province 637000 People’s Republic of China; 2 North Sichuan Medical College, Nanchong, China; 3 Guangyuan Central Hospital, Sichuan, China; 4 Tianjin Key Laboratory of Medical Epigenetics, Key Laboratory of Breast Cancer Prevention and Therapy (Ministry of Education), Department of Biochemistry and Molecular Biology, Tianjin Medical University, Tianjin, China

**Keywords:** Cancer, Computational biology and bioinformatics, Genetics, Immunology, Mathematics and computing

## Abstract

Lung adenocarcinoma (LUAD) is the most frequent subtype of lung cancer globally. However, the survival rate of lung adenocarcinoma patients remains low. Immune checkpoints and long noncoding RNAs are emerging as vital tools for predicting the immunotherapeutic response and outcomes of patients with lung adenocarcinoma. It is critical to identify lncRNAs associated with immune checkpoints in lung adenocarcinoma patients. In this study, immune checkpoint-related lncRNAs (IClncRNAs) were analysed and identified by coexpression. Based on the immune checkpoint-related lncRNAs, we divided patients with lung adenocarcinoma into two clusters and constructed a risk model. Kaplan–Meier analysis, Gene Set Enrichment Analysis, and nomogram analysis of the 2 clusters and the risk model were performed. Finally, the potential immunotherapeutic prediction value of this model was discussed. The risk model consisting of 6 immune checkpoint-related lncRNAs was an independent predictor of survival. Through regrouping the patients with this model, we can distinguish between them more effectively in terms of their immunotherapeutic response, tumour microenvironment, and chemotherapy response. This risk model based on immune checkpoint-based lncRNAs may have an excellent clinical value for predicting the immunotherapeutic response and outcomes of patients with LUAD.

## Introduction

Lung adenocarcinoma (LUAD) is one of the most common subtypes of lung cancer and it ranks first in cancer-related death^1^. Despite the reported efficacy of surgical techniques, radiotherapy, and chemotherapy as treatments for LUAD, the survival of patients with LUAD is still unfavourable^2,3^. In the past decade, immune checkpoints, mainly including PD-1, PD-L1, and CTLA-4, have become a promising and effective treatment strategy capable of significantly prolonging the survival of LUAD patients^4^. However, few biomarkers can predict the efficacy of anti-PD-1/PD-L1/CTLA-4 immunotherapy and stratify the LUAD population by its benefits. Therefore, identifying immune checkpoint-related biomarkers is pivotal for improving the therapy and prognosis of LUAD.

Programmed cell death protein 1 (PD-1), which can bind to its ligand, programmed cell death ligand 1 (PD-L1), is expressed by activated T cells^5,6^. The interactions of PD-L1 on tumour cells with PD-1 signalling have proven to be a potent mechanism to counter the activation of T cells during their escape from host immune responses, which elicits a vitally crucial role in an antitumor immune response^7–9^. Therefore, PD-1/PD-L1 inhibitors (nivolumab and pembrolizumab) were first approved by the US Food and Drug Administration (FDA) for treating melanoma^10^ and renal cell carcinoma^11^ and have also been confirmed to be a significant clinical advance for patients with lung cancer^12–14^. Cytotoxic T lymphocyte-associated protein 4 (CTLA-4) belongs to the CD28 receptor family, which was identified to be activated on the exterior of conventional T cells and attenuate tumour cell proliferation by inhibiting T-cell proliferation and IL-2 secretion^15^. The FDA also approved anti-CTLA-4 inhibitors (ipilimumab) for melanoma^16,17^, non-small cell lung cancer^18^, and other incurable tumours^19,20^.

Long noncoding RNAs (lncRNAs) that exceed two hundred nucleotides in length regulate diverse biological processes and cellular functions^21^. Accumulating studies have revealed that lncRNAs regulate tumour aggression, metastasis, treatment sensitivity, and prognosis by affecting immune cell lineages^22–25^. Although immune checkpoint therapies have been developed as effective therapeutic strategies in tumour immune evasion, no research has yet been carried out on analysing the application values of lncRNAs that act as immune regulators for LUAD clinical immunotherapy.

Our study is the first to develop and validate the IClncRNA-related signature of LUAD by identifying IClncRNAs based on Pearson correlation analysis of data from TCGA-LUAD. Then, we revealed the interaction with tumour-infiltrating immune cells and tumour microenvironment scores and examined the treatment response of LUAD patients to immunotherapy and chemotherapy. Overall, our work developed a new signature that can contribute to immunotherapeutic strategies for treating patients with LUAD.

## Materials and methods

### Data processing

We downloaded the RNA-seq data (FPKM) and the corresponding clinicopathological characteristics from the TCGA-LUAD database (https://cancergenome.nih.gov/)^26^. After screening for data quality, 54 healthy lung and 464 LUAD tissues were selected from individuals with an OS longer than 1 months (Table 1). The whole research process displayed by sup_figure.

**Table 1:** Clinical variables of LUAD patients from TCGA.

Datasets	Training cohort	Testing cohort	Entire cohort
**Age (n)**			
< 65 years	108	107	215
≥ 65 years	124	125	249
**Censor (n)**			
Dead	91	85	176
Alive	141	147	288
**Gender (n)**			
Male	106	104	210
Female	126	128	254
**Stage**			
I	116	139	255
II	57	50	107
III	43	31	74
IV	12	12	24
**T classification**			
T1	73	85	158
T2	147	121	268
T3	24	15	39
T4	9	9	18
**M classification**			
M0	156	156	312
M1	13	11	24
**N classification**			
N0	146	155	301
N1	46	40	86
N2	35	29	64
N3	1	1	2
**Follow-up time**			
< 5 years	207	207	414
≥ 5 years	25	25	50

### Selection of immune checkpoint genes and IClncRNAs

The "limma" package extracted the expression data of the lncRNAs and PD1, PD-L1, and CTLA4 from the LUAD expression profile. Next, we identified IClncRNAs by Pearson correlation analysis, and a total of 75 IClncRNAs were found. The screening criteria were as follows: | Pearson R|> 0.4 and *P* values less than 0.001.

### Identification of LUAD subtypes

Univariate Cox regression models were used to develop the most relevant IClncRNAs for OS of LUAD patients under the R package "survival." We performed nonnegative matrix factorization (NMF) clustering to analyse IClncRNAs associated with significant prognostic value^27^. Using the R package "NMF" on the gene expression matrix, unsupervised NMF clustering procedures were executed, and the optimal cluster number was calculated based on a coexistence correlation coefficient K = 2.

### Construction and verification of the IClncRNA-related model

All LUAD patients were randomly stratified into a training (n = 232) or testing (n = 232) cohort. The training group was used to identify prognostic IClncRNA-related signatures, while the testing and the entire group were used to validate its prognostic value. First, based on the IClncRNAs, we screened IClncRNAs in the training set and further screened lncRNAs distinctly related to overall survival (OS) using the R package "glmnet" impinged on a least absolute shrinkage and selection operator (LASSO)-penalized Cox regression analysis. Eventually, IClncRNAs with OS values were incorporated into the established model. The following formula was computed: risk score (RS) = ΣNi = 1 (lncRNA Exp × coefi), where coefi means the coefficients, and N is the number of lncRNAs. Next, the corresponding risk scores were used to validate the patients in the test set and the entire cohort.

### Assessment of the IClncRNA-related model

Independent prognostic factor analysis of the contributions of each clinical variable and the IClncRNA-related signature was conducted through univariate and multivariate Cox regression analyses. Statistical significance was defined as a P value < 0.05. The nomograms of three clinical features and risk scores were analysed with the “rms” R package to show the predicted survival probabilities for the 1-, 3- and 5-year survival rates of LUAD patients with IClncRNA-related signatures. A calibration plot was then carried out to determine the nomogram accuracy.

### Analysis of tumour immune infiltration

To estimate the relationship between the immune infiltration landscape and the risk score, we utilized the "CIBERSORT" algorithm^28,29^ to estimate the fraction of 22 immune cell types among the LUAD samples, and Spearman correlation was used to assess the relevance between signature-related lncRNAs and immune cells. A P value less than 0.05 was considered significant.

### Gene set enrichment analysis

By using gene set enrichment analysis (GSEA)^30,31^, enrichment analyses were conducted to explore the potential mechanisms and functions between Cluster 1 and Cluster 2, with the following parameters: nPerm = 1000, minGSSize = 10, maxGSSize = 1000, and nominal *P value* < 0.05.

### Immunotherapy and chemotherapy

We implemented the Tumour Immune Dysfunction and Exclusion (TIDE) score, which has proven to be an effective predictor of the ICI therapeutic response^32^. In addition, the "pRRophetic" R package has been utilized in studies evaluating drug sensitivity in cancers by calculating each LUAD sample's IC50 value based on the GDSC website^33–35^.

### Statistical analyses

All statistical analyses were carried out with R software (version 3.6.1). Patients were randomly grouped using the “caret” R package. Univariate and multivariate Cox regression models were used to evaluate the prognostic significance. Kaplan–Meier curves were plotted to analyse the OS between different groups by the log-rank test. The prediction accuracy of the IClncRNA-related risk model was determined by ROC curve analysis. The above statistical analysis was regarded as significantly different at P value < 0.05.

### Ethics statement

We obtained RNA sequence transcriptome data and relevant clinical information of the LUAD patients from the TCGA (https://cancergenome.nih.gov/) database. Their use did not require ethical approval.

## Results

### Identification of IClncRNAs in LUAD individuals

**Figure 1:**
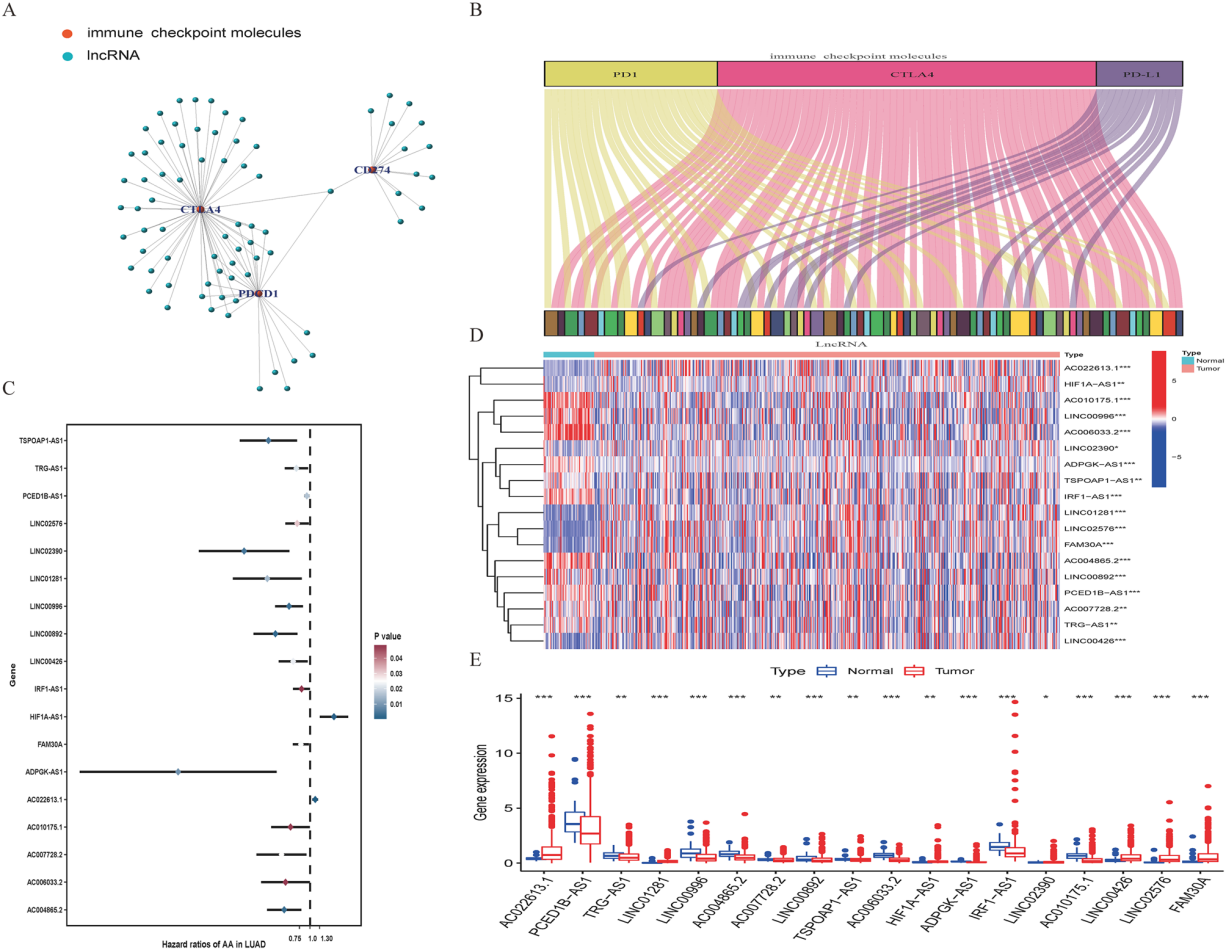
Identification of IClncRNAs of prognostic value in patients with LUAD. (**A**) Network diagram for PD1, PD-L1, CTLA4 and 75 IClncRNAs. (**B**) Sankey relational diagram for three immune checkpoint genes and the IClncRNAs. (**C**) Univariate Cox regression analysis revealed that the 18 selected lncRNAs were significantly correlated with the clinical prognosis. (**D**) Heatmap for the expression difference of IClncRNAs between tumour and normal tissue. (**E**) Boxplot for the expression difference of IClncRNAs between tumour and normal tissue (*P < 0.05, **P < 0.01, ***P < 0.001, ****P < 0.0001).

We abstracted 14,086 lncRNAs from the TCGA-LUAD samples associated with immune checkpoint genes (PD1, PD-L1, and CTLA4) by Pearson correlations, and 75 lncRNAs were screened as IClncRNAs that are visualized in Fig. 1A,B. Merging the survival information with the IClncRNAs from 464 LUAD patients, 18 prognostic IClncRNAs were identified by univariate Cox regression analysis (Fig. 1C; *P* < 0.05), all considered protective factors except HIF1A-AS1 and AC022813.1. As shown in Fig. 1D,E, the expression of 18 IClncRNAs showed significant differences between normal and LUAD tissues (*P* < 0.05).

### Classification of LUAD by IClncRNAs

**Figure 2:**
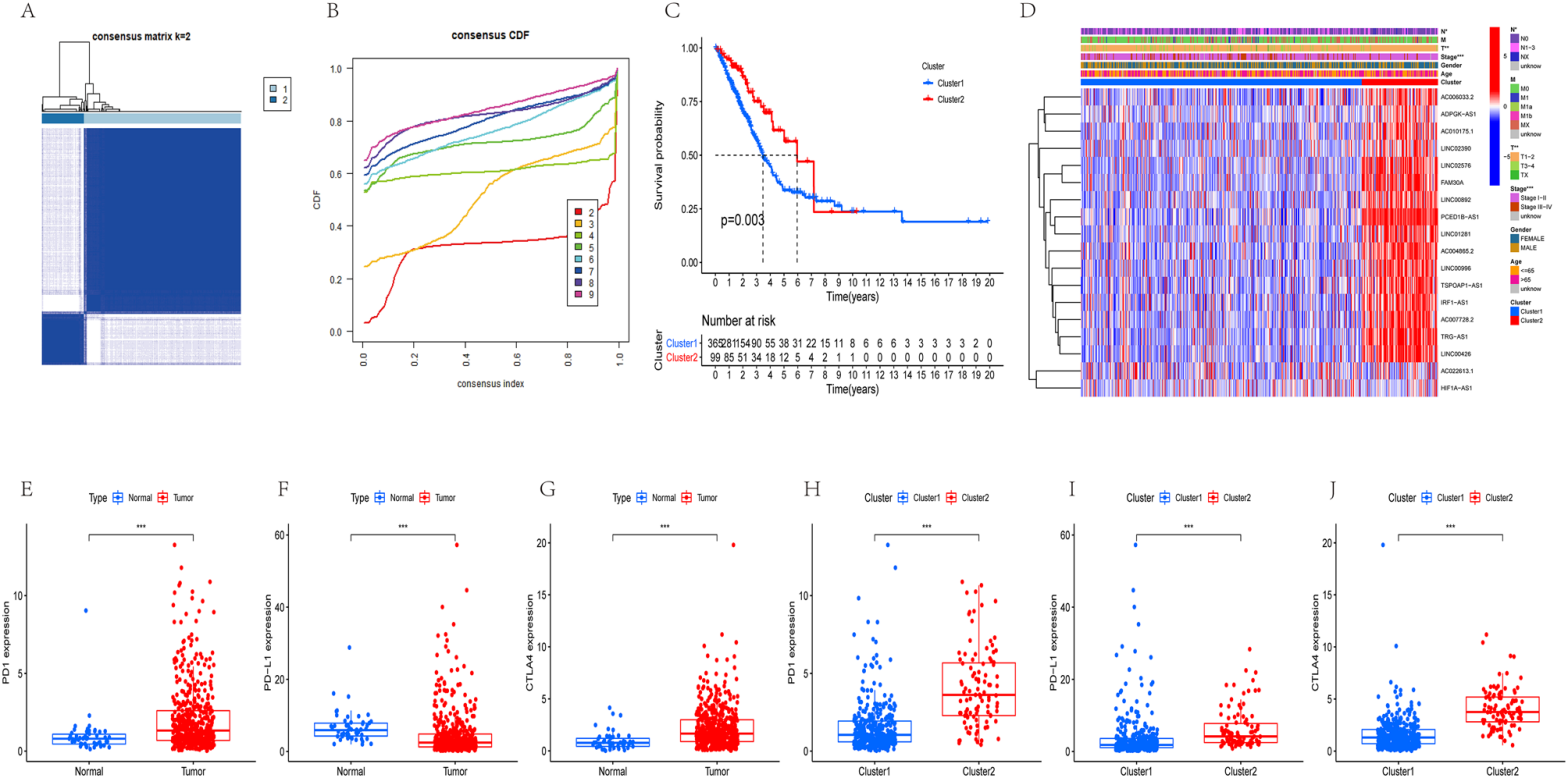
Identification of LUAD subtypes using NMF consensus clustering in LUAD patients. (**A**,**B**) NMF clustering using 18 IClncRNAs. The patients were divided into two clusters (Cluster 1 and Cluster 2). (**C**) Survival analysis of patients in Clusters 1 and 2 in the LUAD cohort. (**D**) Heatmap of two clusters defined by IClncRNA expression. (**E**–**G**) Differences in PD1, PD-L1, and CTLA4 expression between lung cancer tissue and normal tissue. (**H**–**J**) Differences in PD1, PD-L1, and CTLA4 expression between Cluster 1 and Cluster 2.

Extracting 18 IClncRNAs expressed in the LUAD sample, we subsequently categorized 464 samples using a consensus clustering algorithm to elucidate their differences between subgroups. We found that k = 2 was the optimum value; thus, patients in the entire cohort were sorted into subtypes (Fig. 2A,B).

The patients were separated into Cluster 1 (n = 365) and Cluster 2 (n = 99). Cluster 1 (C1) had significantly worse OS than Cluster 2 (C2) (Fig. 2C). The heatmap showed the differences in IClncRNA expression between subgroups (Fig. 2D), and the majority of the IClncRNAs were overexpressed in Cluster 2. Clinical variables, such as N, T, and stage, differed between the clusters. In addition, PD1 and CTLA4 expression in LUAD were higher than in normal samples, while PD-L1 had lower expression in LUAD (Fig. 2E–G). Similarly, we found significantly higher PD1, PD-L1, and CTLA4 expression in C2 (Fig. 2H–J).

### Immune cell infiltration and functional enrichment analysis between C1 and C2

**Figure 3:**
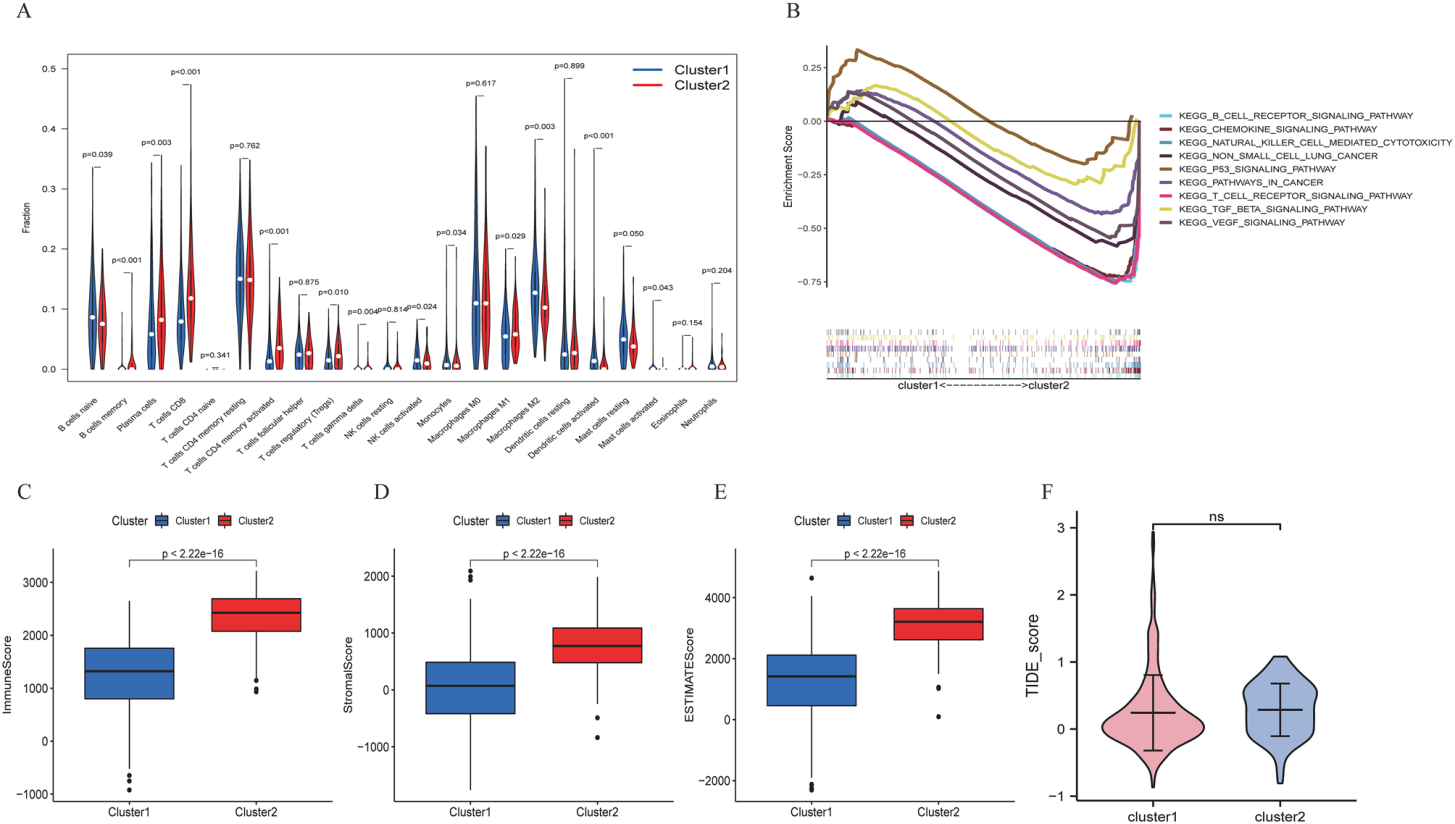
Identification of the immune cell infiltration landscape, tumour microenvironment score, KEGG pathway, and TIDE score in the two clusters. (**A**) The difference in immune cell infiltration between Cluster 1 and Cluster 2. (**B**) KEGG pathway analysis between Cluster 1 and Cluster 2. (**C**–**E**) ImmuneScore, StromalScore and ESTIMATEScore between Cluster 1 and Cluster 2. (**F**) The difference in TIDE_score between the two clusters.

Then, we explored the association with the TME between the two clusters based on CIBERSORT. The violin plot results indicated that there were 13 immune infiltrating cell differences between clusters (Fig. 3A), with memory B cells, plasma cells, CD8 T cells, activated memory CD4 T cells, regulatory T cells (Tregs), and M1 macrophages having higher infiltration in Cluster 2, while native B cells, gamma delta T cells, activated NK cells, M2 macrophages and activated mast cells had more infiltration in Cluster 1 (P < 0.05). The TME scores in Cluster 1 were considerably higher than those in Cluster 2 (Fig. 3C–E; p < 0.05). However, the response rate to ICIs predicted by the TIDE score showed no difference between Clusters 1 and 2 (Fig. 3F). In addition, to predict the functions or pathways involved in IClncRNAs from LUAD, GSEA was selected for comparison between the clusters. The results were highly enriched in Cluster 2, including the non-small cell lung cancer pathway, T-cell receptor signalling pathway, B-cell receptor signalling pathway, NK-cell-mediated cytotoxicity, and VEGF signalling pathway (P < 0.05; Fig. 3B), and the unwarping IClncRNAs proved to be remarkably associated with the immune status of patients in the TCGA-LUAD cohort.

### Construction and validation of the IClncRNA-associated risk model in LUAD patients

**Figure 4:**
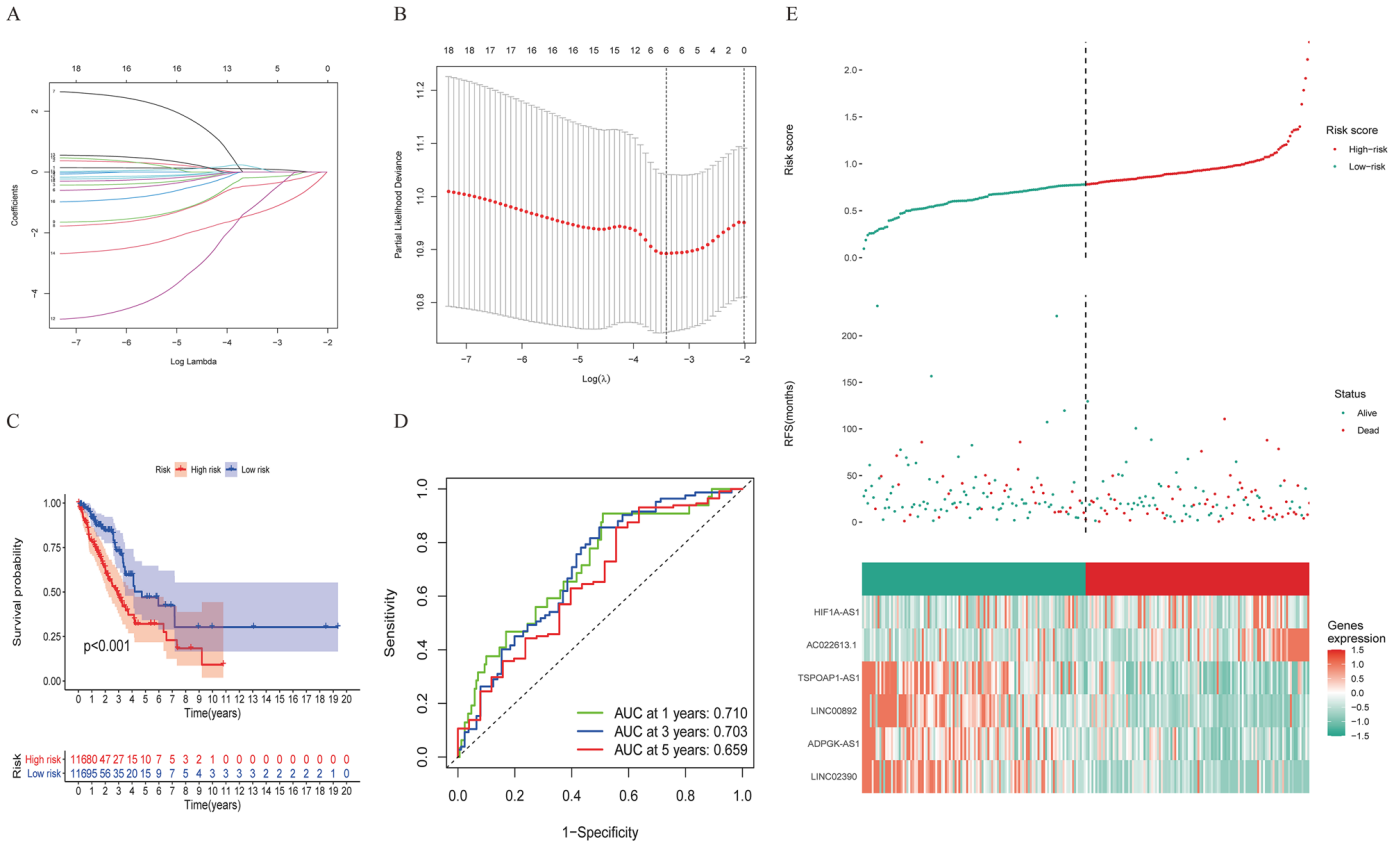
A risk model for LUAD patients based on IClncRNAs. (**A**) The LASSO coefficient profile of 18 OS-related lncRNAs. The perpendicular imaginary line is drawn at the value chosen by tenfold cross-validation. (**B**) The tuning parameters (log λ) of OS-related proteins were selected to cross-verify the error curve. According to the minimal criterion and 1-se criterion, the perpendicular imaginary line is drawn at the optimal value. (**C**) Kaplan–Meier survival curves of OS of patients in the high- and low-risk groups. (**D**) The time‐dependent ROC analyses of this model in the training cohort. (**E**) The distribution of risk scores based on the IClncRNAs, vital statuses of the patients sorted by risk score and the six-lncRNA expression heatmap in the training cohort.

The results of the univariate Cox regression model showed that 18 out of the 75 IClncRNAs were significantly associated with the overall survival of patients with LUAD. Based on the Lasso Cox regression model, these 18 lncRNAs were screened out to avoid overfitting, improve the accuracy and obtain the best penalty parameters. Hence, 6 IClncRNAs (AC022613.1, LINC00892, TSPOAP1-AS1, HIF1A-AS1, ADPGK-AS1, and LINC02390, Table 2) were eventually used to construct the IClncRNA-associated signature (Fig. 4A,B). K-M survival analysis showed worse OS in the high-risk group (Fig. 4C). The ROC curves showed that our signature had a robust predictive ability, with AUCs predicting 1-year, 3-year, and 5-year overall survival of 0.710, 0.703, and 0.659, respectively (Fig. 4D). Next, we ranked the training cohort by the risk score from low to high; the follow-up time and genetic heatmap of the population are also shown by this standard (Fig. 4E). The heatmaps showed that LINC00892, TSPOAP1-AS1, ADPGK-AS1, and LINC02390 expression in the low-risk group was significantly greater than that in the high-risk group, while the other lncRNAs were downregulated (Fig. 4E).

**Table 2:** The model information of lung adenocarcinoma.

Gene	Coef
AC022613.1	0.101634
LINC00892	− 0.42989
TSPOAP1-AS1	− 0.18157
HIF1A-AS1	0.1437
ADPGK-AS1	− 1.05709
LINC02390	− 1.35457

**Figure 5:**
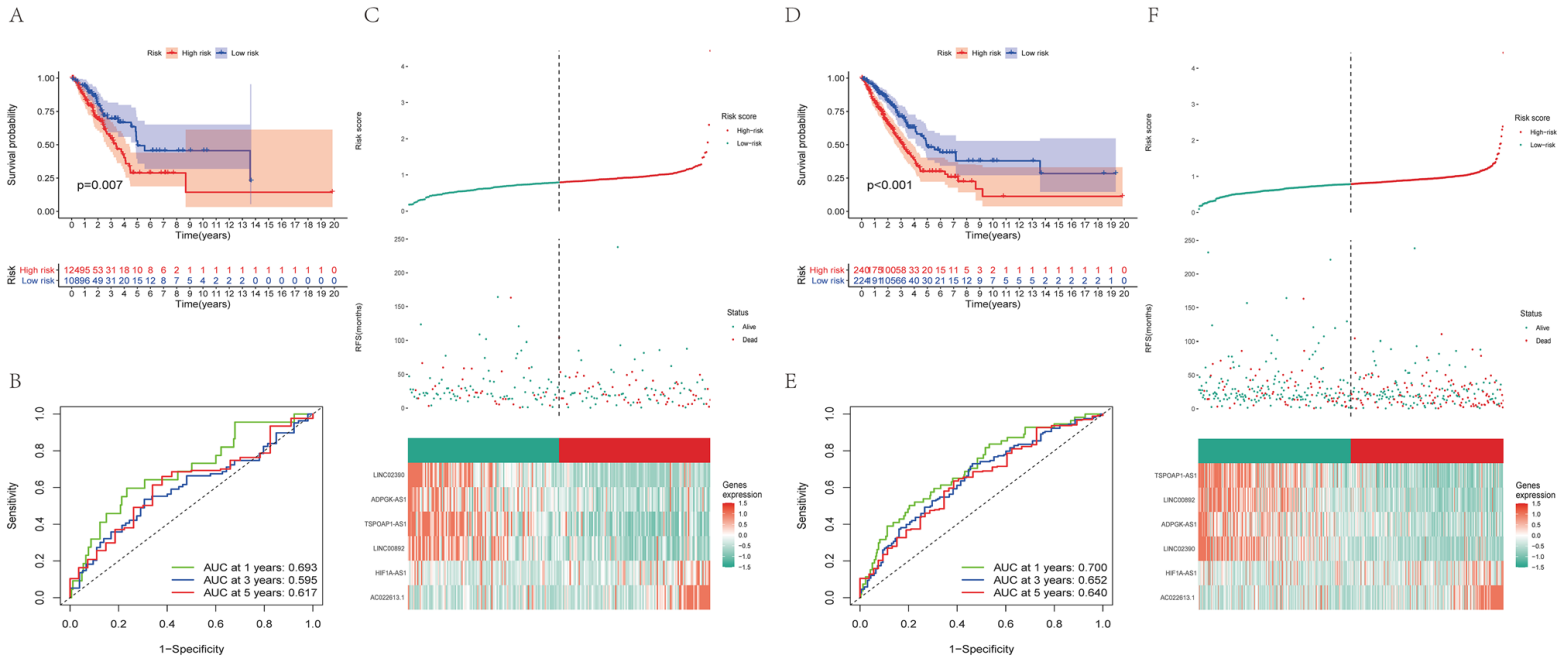
The prognostic value of the risk model of the six IClncRNAs in the TCGA testing and entire cohorts. (**A**) Kaplan–Meier survival curves of OS of patients in the high- and low-risk groups for the testing cohort. (**B**) The time‐dependent ROC analyses of this model in the testing cohort. (**C**) The distribution of risk scores based on the IClncRNAs, vital statuses of patients sorted by risk score and the six-lncRNA expression heatmap in the testing cohort. (**D**) Kaplan–Meier survival curves of OS of patients in the high- and low-risk groups for the entire cohort. (**E**) The time‐dependent ROC analyses of this model in the entire cohort. (**F**) The distribution of risk scores based on the IClncRNAs, vital statuses of patients sorted by risk score and the six-lncRNA expression heatmap in the entire cohort.

To further examine the predictive efficacy of the model, the IClncRNA-associated risk model was verified in the testing and entire groups. We calculated the optimal cut-off point and randomly separated patients into low-risk and high-risk groups. The survival analysis showed significantly longer OS in the high-risk group than in the low-risk group (P < 0.05; Fig. 5A,D). As shown in Fig. 5B, all of the time‐dependent ROC curve results obtained superior AUC values for the 1-year, 3-year, and 5-year OS of LUAD patients (AUC = 0.693, 0.595, and 0.617). The AUCs in the entire set for predicting patient OS at 1, 3 and 5 years were 0.700, 0.652 and 0.640 (Fig. 5E), respectively. The six-lncRNA expression heatmap sorted by the risk score is also shown in Figs. 5C,F.

**Figure 6:**
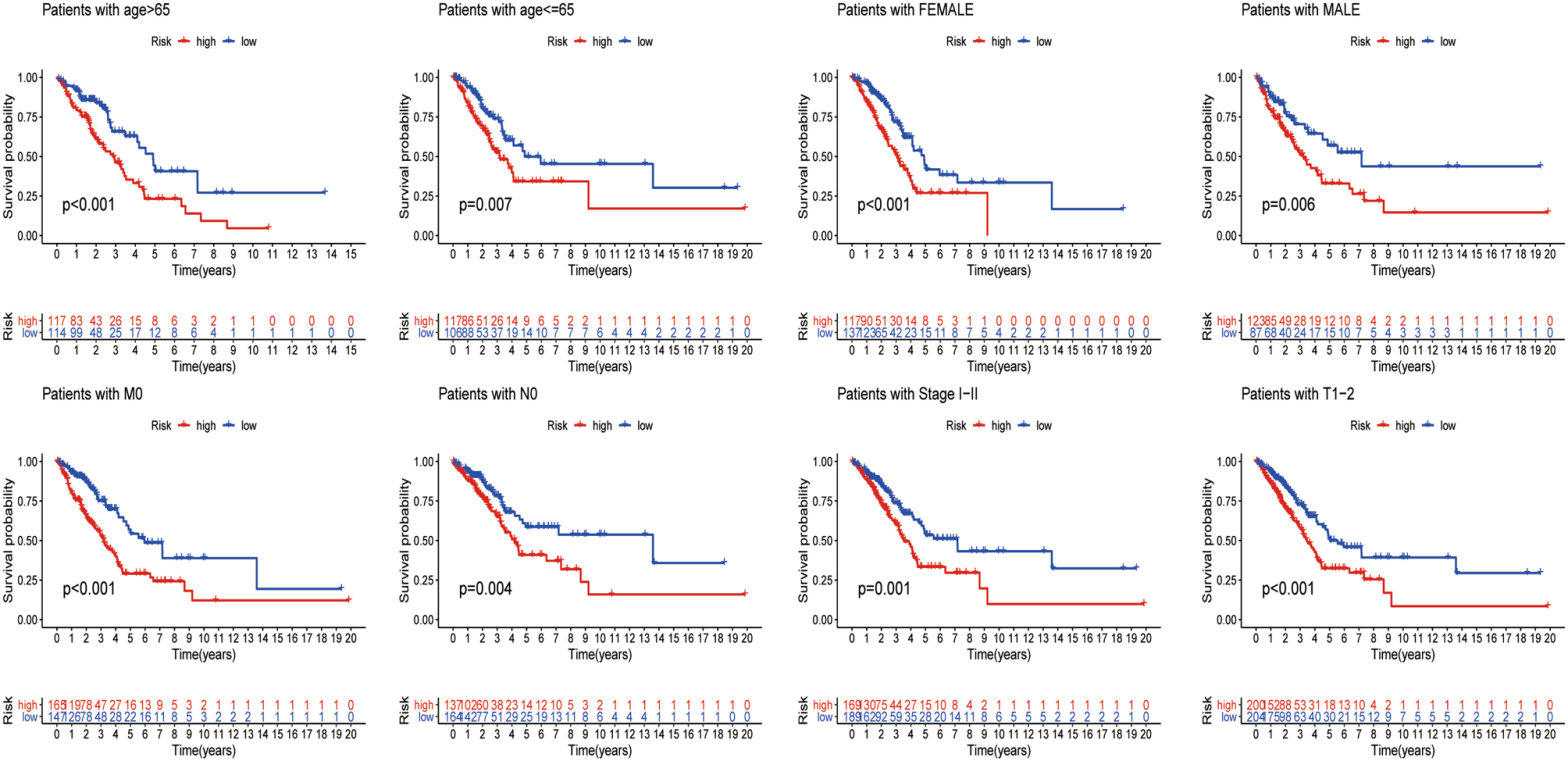
Kaplan–Meier curves of differences in overall survival stratified by sex, age, or TNM stage between the low-risk and high-risk groups in the entire TCGA cohort.

Stratified survival analysis by the universal clinicopathologic characteristics, gender, age, stage, or tumour stage subgroups in low-risk group patients was significantly unfavourable to the low-risk group (P < 0.05; Fig. 6).

### The IClncRNA-related risk score as an independent risk factor

**Figure 7:**
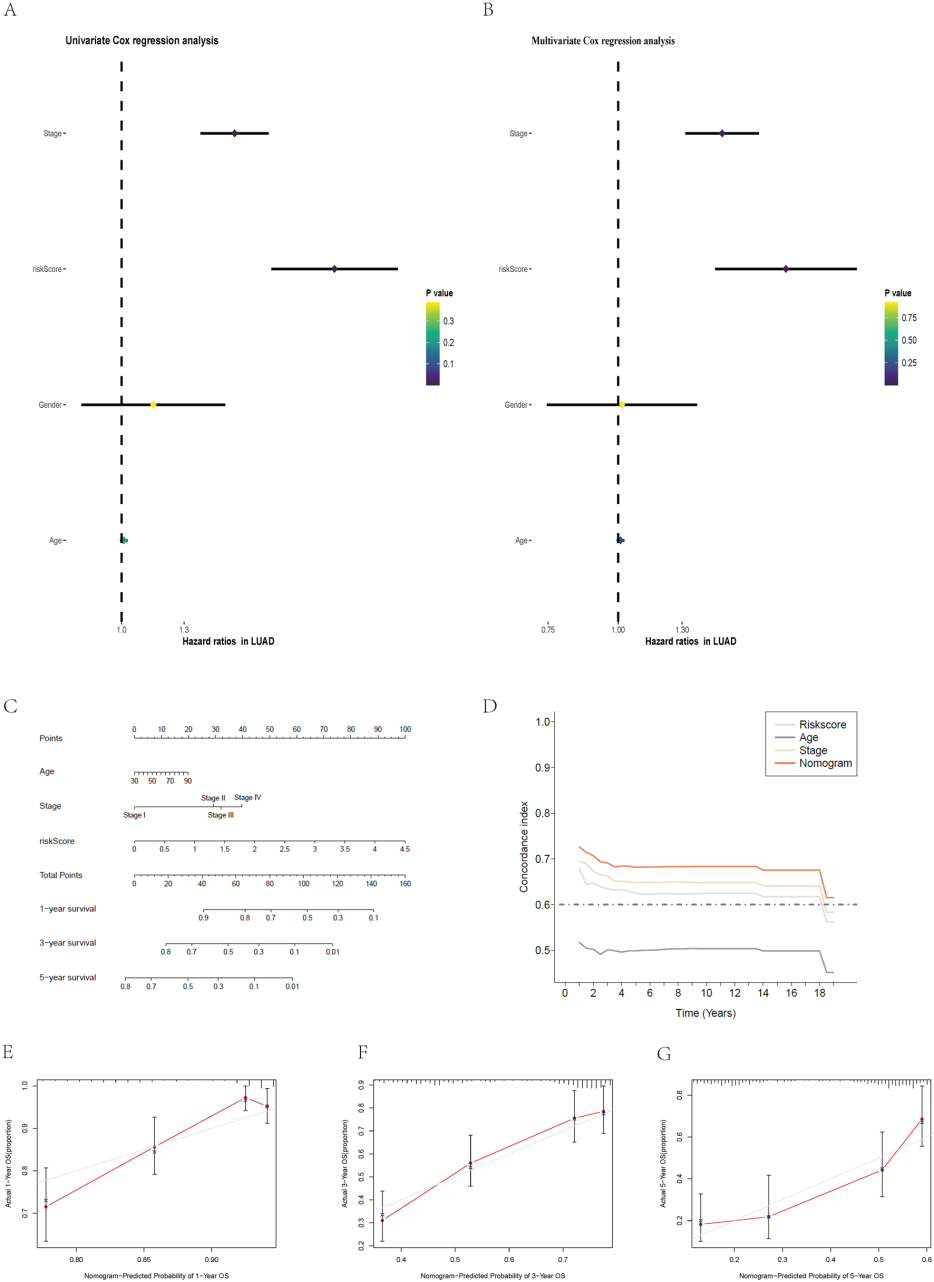
Assessment of the prognostic risk model and clinical characteristics in the entire cohort; construction and evaluation of a prognostic nomogram. (**A**,**B**) Univariate and multivariate analyses of the clinical characteristics and risk level with OS. (**C**) The nomogram predicts the probability of 1-, 3-, and 5-year OS. (**D**) Concordance indices of the risk score and clinical characteristics. (**E**–**G**) The nomogram's calibration plot predicts the probability of 1-, 3-, and 5-year OS.

To evaluate whether this risk model of IClncRNAs had independent prognostic characteristics, stage and risk scores were strongly associated with prognosis via univariate and multivariate analyses, for which the HRs of the risk score were 2.449 and 1.990 (p < 0.001; Fig. 7A,B), indicating that the signature was an independent prognostic factor for LUAD.

### Construction and evaluation of the nomogram

We then constructed a nomogram with the IClncRNA risk score and other clinicopathological variables to predict the 1-, 3-, and 5-year survival of LUAD patients (Fig. 7C). The concordance index of the risk grade was always higher than that of the other clinical factors, indicating a promising prognostic ability (Fig. 7D). Moreover, the calibration plot was identified as a sensitive prediction strategy for the prognosis of LUAD patients (Fig. 7E–G).

### Clinical correlation analysis between the tumour immune microenvironment and risk model

**Figure 8:**
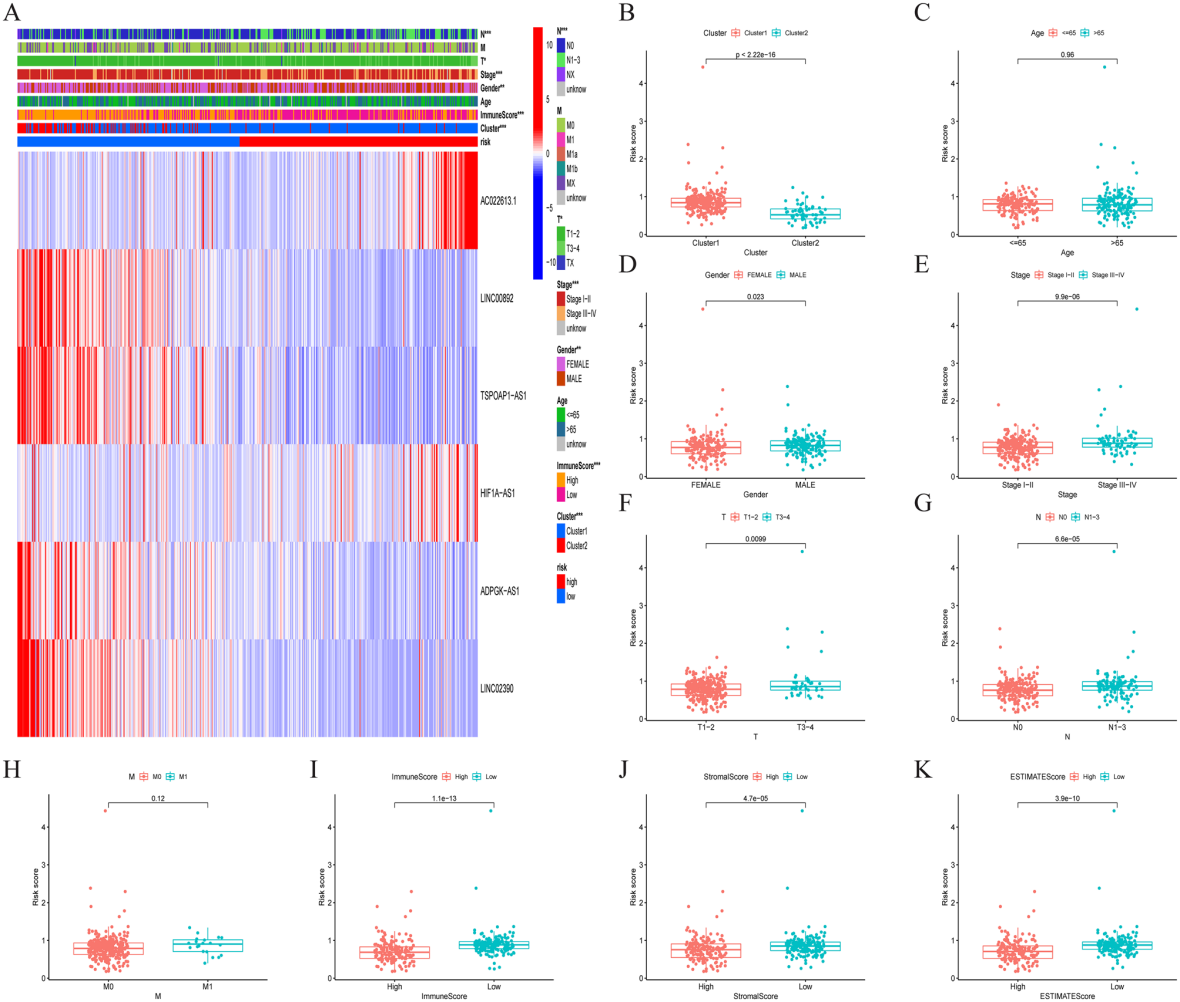
Estimation of the tumour immune microenvironment and clinical characteristics with the IClncRNA model in the entire TCGA cohort. (**A**) Heatmap of high- and low-risk groups defined by IClncRNA expression. (**B**–**H**) The relationship between the risk score and clinical characteristics. (**I**–**K**) Differences in the risk scores in the high and low tumour microenvironment score groups (ImmuneScore, StromalScore, and ESTIMATEScore).

We further explored the relationship between risk scores and clinical characteristics. The outcomes demonstrated that the expression levels of the 4 IClncRNAs were high in the low-risk group (Fig. 8A). We also analysed the risk score in different LUAD subgroups (Fig. 8A) to explore their correlations with clinicopathological features in LUAD, among which age and metastasis were not significantly different between the risk groups. At the same time, there was a significant difference in sex and stage (Fig. 8B–G). Compared to the low-risk group, the results of the stage and three types of TME scores were greater for patients in the high-risk group (Fig. 8H–K), which implies a potential correlation between the model and the LUAD microenvironment.

### Therapeutic response assessment

**Figure 9:**
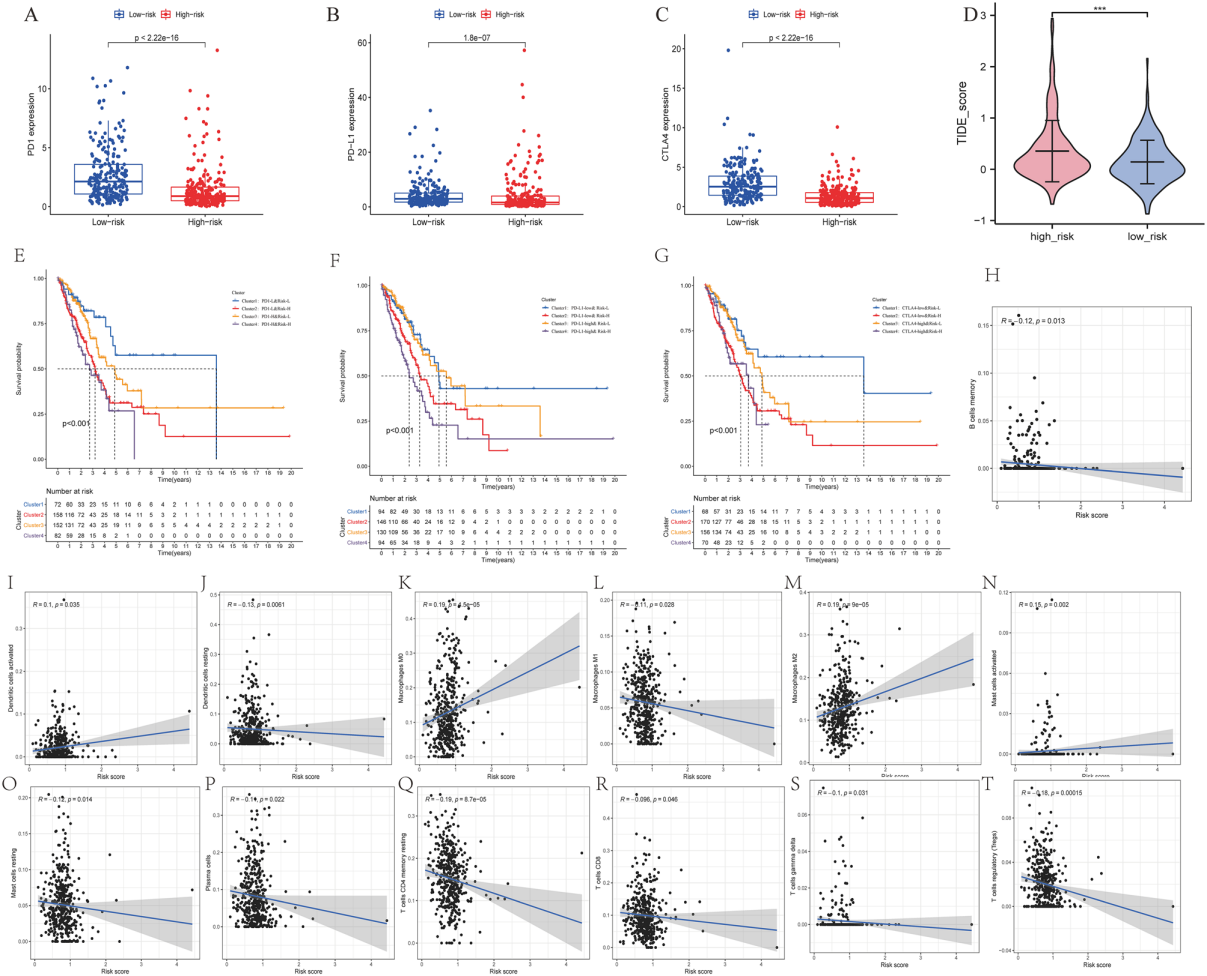
Estimation of immune checkpoint gene expression and cancer immunotherapy response with the model in the entire TCGA cohort. (**A**–**C**) Boxplot showing the differences in PD1, PD-L1, and CTLA4 expression between the high- and low-risk groups. (**D**) TIDE prediction score between the high- and low-risk patients. (**D**) Kaplan–Meier survival curves of the four patient groups stratified by the IClncRNA model and PD-1 (**E**), PD-L1 (**F**), and CTLA-4 (**G**). (**H**–**T**) Correlation between the risk score and immune cells.

We then explored the differences in PD1, PD-L1, and CTLA4 expression between the IClncRNA model and ICI biomarkers. The response to immunotherapy was better in patients in the low-risk group (P < 0.05; Fig. 9A–C). Intriguingly, TIDE, which has emerged as a vital predictive immunotherapeutic biomarker^36^, had significantly lower scores in the low-risk group than in the high-risk group, indicating that our signature could better predict the response of LUAD to immunotherapy (p < 0.05; Fig. 9D).

The IPS has promising potential for cancer patients treated with CTLA-4 and PD-1 blockers^37^. In our research, the patients with high-risk scores/PD-1 negative, high-risk scores/PD-1 positive were found to have worse survival than those with low-risk scores/PD-1 positive and low-risk scores/PD-1 negative (p < 0.05; Fig. 9E). Similarly, PD-L1 or CTLA-4 stratification in patients with a risk score showed the same survival pattern following the PD-1 trend (p < 0.05; Fig. 9F,G), which indicated that the high-risk group patients showed a better opportunity for ICI application. After discussing the immunotherapy possibility of the signature, we investigated the risk score’s links to immune infiltration. As shown in Fig. 9H–T, it was inversely correlated with the abundance of memory B cells, resting dendritic cells, M1 macrophages, resting mast cells, plasma cells, CD8 T cells, and regulatory T cells (Tregs) (p < 0.05), whereas it had the same trend as activated dendritic cells and activated M0 macrophages (p < 0.05). These observations indicate that the IClncRNA-related signature could predict the efficacy of immune checkpoint inhibitors for LUAD.

**Figure 10:**
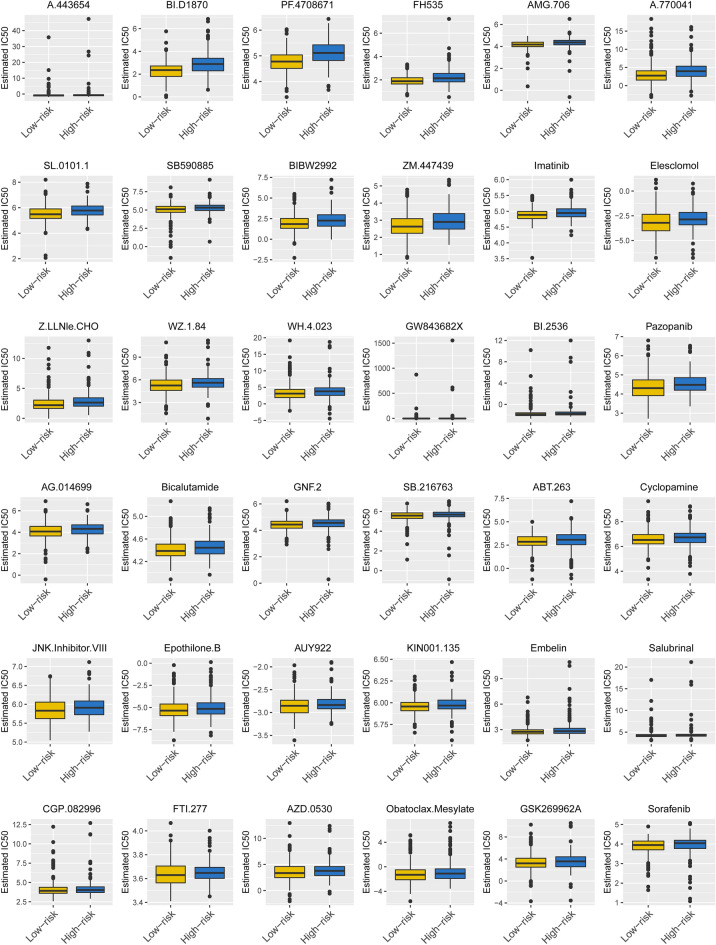
Different chemotherapeutic responses in high- and low-risk patients with LUAD (*P* < 0.05). sup_figure: Study flow chart.

Apart from the above analysis, we explored the resistance to chemotherapy changes to estimate the IC50 between the two risk groups to investigate whether this signature also had a chemotherapeutic value. The results revealed significant differences in 36 targeting drugs between the groups, and patients in the high-risk group had strong drug sensitivity (P < 0.05; Fig. 10), suggesting it also has utility in predicting the response to chemotherapy.

## Discussion

LUAD is the most common subtype of lung cancer that threatens human health^3^. Despite advances in therapeutic strategies such as surgical techniques, radiotherapy, and chemotherapy, the survival of patients with LUAD is still unfavourable. Therapies targeting immune checkpoints that mainly involve PD1, PD-L1, and CTLA4 have been widely applied in the treatment of advanced cancers, including melanoma^38^ and non-small cell lung cancer^39–41^. However, there are some limitations regarding immune checkpoints: their expression level does not directly reflect the tumour’s sensitivity to immunotherapy or the OS^42^. LncRNAs, a large class of noncoding RNAs > 200 nucleotides (nt) in length, have recently received increasing attention. They have been reported to function as essential regulators in tumour-infiltrating immune cells^24,43–45^. However, little is known about the roles of IClncRNAs in immunity assessment and immunotherapeutic responses in LUAD.

Using data from the TCGA-LUAD dataset, we first identified 18 of 75 IClncRNAs that had confirmed prognostic value. Moreover, we categorized the patients into two clusters by consensus clustering analysis to explore the immune checkpoint-related subtypes of LUAD. The results showed that tumour stage, OS, immune checkpoint expression, immune cell infiltration, and tumour microenvironment score exhibited significant differences between the clusters. However, there was no significant difference in TIDE, which is employed to evaluate the immunotherapy response.

In our risk model, we identified a signature of six IClncRNAs associated with OS that was constructed by multivariate regression analysis. Researchers previously found that LINC00892 is associated with the tumour microenvironment and immunotherapy response in bladder cancer^46^. These results are consistent with ours. The long noncoding RNA TSPOAP1-AS1 is a potential diagnostic biomarker for paediatric septic shock^47^. Other studies have shown that the long noncoding RNA TSPOAP1-AS1 is associated with obesity^48^ and influenza A virus replication^43^. Consistently, researchers previously reported that TSPOAP1-AS1 is a prognostic biomarker for pancreatic cancer. HIF1a-AS1 is involved in many types of malignant tumours^49^ and it plays a vital role in liver fibrosis^47^. Inhibition of HIF1a-AS1 could promote apoptosis of hepatoma cells induced by starvation^50^. These results are consistent with ours. Long noncoding RNA ADPGK-AS1 was associated with a poor prognosis of osteosarcoma^51^, breast cancer^52^, pancreatic cancer^53^, and gastric cancer^53^, and was also related to molecular subtypes of prostate cancer^54^. These results indicate that ADPGK-AS1 could play an essential role in regulating the occurrence and development of many cancers. Its mechanism is worthy of further study. Reports on the long noncoding RNAs LINC02390 and AC022613.1 are rare and they are worthy of further study.

Based on the above IClncRNAs, the IClncRNA-related signature that was constructed in the training population was validated successfully in the testing and entire set. The risk scoring model had good prediction effectiveness and was an independent risk factor in multivariate Cox regression analysis. The associated nomogram showed perfect consistency for 1-year, 3-year, and 5-year OS. Patients with a high-risk score in all three risk cohorts had significantly worse OS rates than the low-risk group. Moreover, we found that not only clinical stages but also tumour-infiltrating immune cells, immune checkpoint gene expression, and the tumour microenvironment score had significantly different distributions between the two risk groups. Above all, our results revealed that patients at high risk have higher TIDE scores, which has been confirmed to predict the efficacy of anti-PD1 and anti-CTLA4 therapy^55^. Therefore, we hypothesize that the prediction model might have massive potential for selecting LUAD patients likely to benefit from immunotherapy.

Some limitations of the current study need to be highlighted. First, due to the limited sample sizes, more large-scale data are warranted for external verification. Second, to sufficiently understand their potential mechanisms, it is necessary to conduct in vitro and in vivo experiments on the identified IClncRNAs.

In summary, we successfully established and verified an IClncRNA-associated signature for predicting the survival of patients with LUAD. This signature based on 6 IClncRNAs was better than two molecular subtype methods in predicting the immunotherapeutic response of LUAD patients, especially the TIDE score prediction. Consequently, this signature in LUAD could offer novel insights into a theoretical foundation for future studies on immune treatment and be helpful for personalized management of LUAD in the clinical environment.

## Supplementary Information

Supplementary Information 1.

## Data Availability

The data are available in a public, open access repository. The datasets analysed during the current study are available in The Cancer Genome Atlas (TCGA) network (https://cancergenome.nih.gov/).
