# Supplementary figures and images for: Evaluating the utility of an immune checkpoint-related lncRNA signature for identifying the prognosis and immunotherapy response of lung adenocarcinoma
Source: Sci Rep. 2022 Jul 27;12:12785. doi: 10.1038/s41598-022-16715-0 (PMC9329438; doi:10.1038/s41598-022-16715-0)

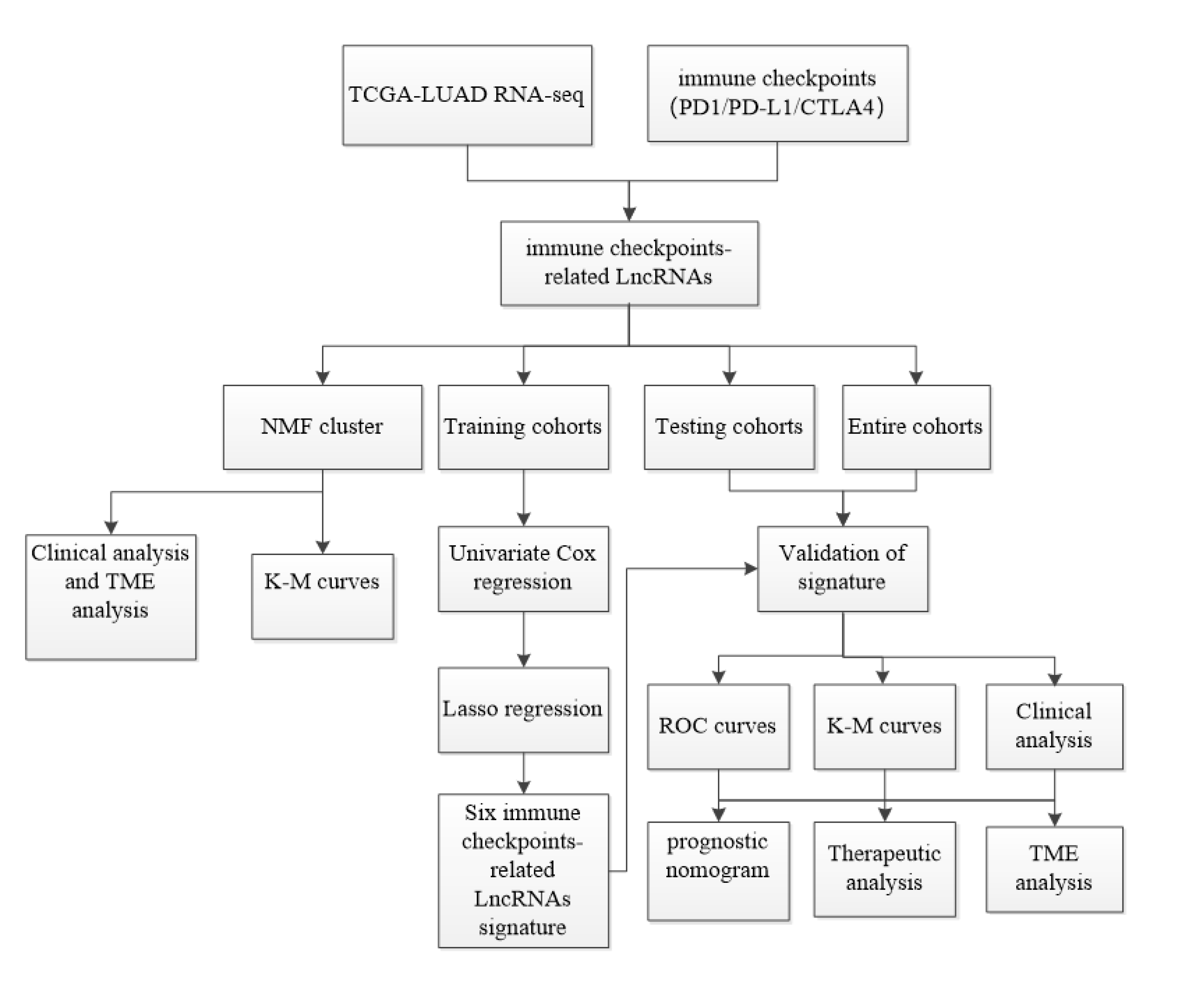

Supplement: Supplementary file 1 — Supplementary Information 1. [file 41598_2022_16715_MOESM1_ESM.tif]
